# Rapid design and implementation of an adaptive pooling workflow for SARS-CoV-2 testing in an NHS diagnostic laboratory: a proof-of-concept study

**DOI:** 10.12688/wellcomeopenres.17226.1

**Published:** 2021-10-13

**Authors:** Michael Crone, Paul Randell, Zoey Herm, Arthi Anand, Saghar Missaghian-Cully, Loren Perelman, Panagiotis Pantelidis, Paul Freemont

**Affiliations:** 1 London Biofoundry, Imperial College Translation and Innovation Hub, White City Campus, 84 Wood Lane, London, W12 0BZ, UK; 2 Section of Structural and Synthetic Biology, Department of Infectious Disease, Imperial College London, London, SW7 2AZ, UK; 3 UK Dementia Research Institute Centre for Care Research and Technology, Imperial College London and the University of Surrey, London, Guildford, UK; 4 Department of Infection and Immunity, North West London Pathology, London, UK; 5 Imperial College Healthcare NHS Trust, Charing Cross Hospital, Fulham Palace Road, London, W6 8RF, UK; 6 Riffyn, Inc., 484 9th Street, Oakland, California, 94607, USA; 7 Histocompatibility and Immunogenetics Laboratories, Department of Infection and Immunity, North West London Pathology, London, UK; 8 Imperial College Healthcare NHS Trust, Hammersmith Hospitals Trust, Du Cane Road, London, W12 0HS, UK; 9 North West London Pathology, London, UK

**Keywords:** laboratory automation, diagnostics, SARS-CoV-2

## Abstract

**Background:** Diagnostic laboratories are currently required to provide routine testing of asymptomatic staff and patients as a part of their clinical screening for SARS-CoV-2 infection. However, these cohorts display very different disease prevalence from symptomatic individuals and testing capacity for asymptomatic screening is often limited. Group testing is frequently proposed as a possible solution to address this; however, proposals neglect the technical and operational feasibility of implementation in a front-line diagnostic laboratory.

**Methods: **Between October and December 2020, as a seven-week proof of concept, we took into account scientific, technical and operational feasibility to design and implement an adaptive pooling strategy in an NHS diagnostic laboratory in London (UK). We assessed the impact of pooling on analytical sensitivity and modelled the impact of prevalence on pooling strategy. We then considered the operational constraints to model the potential gains in capacity and the requirements for additional staff and infrastructure. Finally, we developed a LIMS-agnostic laboratory automation workflow and software solution and tested the technical feasibility of our adaptive pooling workflow.

**Results: **First, we determined the analytical sensitivity of the implemented SARS-CoV-2 assay to be 250 copies/mL. We then determined that, in a setting with limited analyser capacity, the testing capacity could be increased by two-fold with pooling, however, in a setting with limited reagents, this could rise to a five-fold increase. These capacity increases could be realized with modest additional resource and staffing requirements whilst utilizing up to 76% fewer plastic consumables and 90% fewer reagents. Finally, we successfully implemented a plate-based pooling workflow and tested 920 patient samples using the reagents that would usually be required to process just 222 samples.

**Conclusions: **Adaptive pooled testing is a scientifically, technically and operationally feasible solution to increase testing capacity in frontline NHS diagnostic laboratories.

## Introduction

The need for SARS CoV-2 testing as part of the pandemic response has placed significant strain on diagnostic services in terms of testing capacity and the availability of reagents and labware. This has provided multiple challenges to both maintain and, at the same time, increase testing capacity. To address this there has been a recent surge in interest to explore the utility of pooling as a strategy to optimise SARS CoV-2 testing ^1– 3^. Initially described during the Second World War for testing soldiers for syphilis ^4^, pooling has since been used in multiple settings, from screening ^5^, to surveillance and diagnostic testing ^6^. One of the simplest strategies, termed Dorfman pooling, involves a 2-step process with initial testing of pool of samples followed by testing individual samples in positive pools ^4^. Further rounds of testing can be added to this strategy to reduce the number of individual tests that are required, but these approaches come at the expense of increasing the time to getting and reporting the results. Other proposals of adaptive ^7– 9^ and non-adaptive pooling ^9– 11^ promise further increases in efficiency and, although often mathematically elegant, neglect to consider the complexities of required laboratory automation and implementation outside of a single, local laboratory context ^9^.

Utilising a pooling strategy for SARS-CoV-2 testing promises the possibility of significantly increasing analyser testing capacity but several limitations have to be considered. These include reduced analytical sensitivity with a possible increase in the reporting of false negative results, a loss in the ability to assess individual sample adequacy, prevalence levels (as pooling is optimal if infection prevalence is low) and the requirement for more complicated laboratory workflows and infrastructure. Due to the scale and rapidity of the SARS-Cov-2 pandemic, many different assays and pooling strategies have been used for SARS CoV-2 testing with some assays having already received regulatory approval ^12, 13^. Furthermore, interim guidance for laboratories on the use of pooling has also been developed by the Centers for Disease Control and Prevention (CDC) ^14^. The CDC describe using pooling to expand SARS CoV-2 nucleic acid diagnostic or screening testing capacity for an FDA approved test and also how to determine prevalence on a local rolling average of the rate of positive tests and communicate the limitations associated with pooled testing. 

In the context of the UK National Testing Programme, Public Health England (PHE) has issued a standard operating procedure for pooling ^15^ to support an increase in testing capacity during a time of low background prevalence, reducing reagent consumption per test and to increase overall total testing capacity in the country. Here, we expand upon this guidance and investigate how pooling could be implemented in the context of an NHS diagnostic laboratory, to understand any potential benefits, while addressing associated technical and operational challenges for implementation in a front-line SARS-CoV-2 testing service.

## Methods

### Determination of analytical sensitivity

Previously characterised and quantified ^16^ MS2 VLPs containing the N-gene (accession number: NC_045512) of SARS-CoV-2 were transferred using an Echo 525 acoustic liquid handler (Labcyte) to create a standard curve from 100 000 to 50 copies/mL for RNA extraction. A sample of 200 μl was used for RNA extraction using the Maxwell HT Viral TNA kit (Promega) on the CyBio FeliX liquid handler (Analytik Jena) with an elution volume of 50 μl. Subsequent RT-qPCR was performed using the TaqPath ^TM^ COVID-19 CE-IVD RT-PCR Kit (ThermoFisher Scientific) according to the manufacturer’s instructions and thermocycled on a qTower3 (Analytik Jena).

### Impact of pooling on capacity

The following assumptions were used for modelling the impact of pooling on capacity. These assumptions were based on timing various steps as well as the time taken during the technical feasibility experiment.

Capacity benefit of the integrated pooling workflow:

$$Increase\;in\;capacity\;=\;\frac{Total\;Number\;of\;Samples}{Number\;of\;tests\;used\;to\;test\;pools+Number\;of\;tests\;used\;for\;retesting}\;(1)$$

### Technical feasibility of pooled testing

North West London Pathology’s Sunquest LIMS system was used to store patient information and associated final positive/negative/inconclusive results. A partitioned test environment of Sunquest was created to allow for feasibility testing of the complete workflow while performing the pilot. A cloud-based ‘command centre’ web app was engineered specifically for SARS-CoV-2 plate-based pooling diagnostics and utilised the Riffyn Nexus ^®^ software platform as a backend data architecture.

In bulk, patient samples were booked into the Sunquest LIMS system. Swabs were removed and samples were then neutralised in Copan ^TM^ tubes with 2.5 mL of MagBead Viral RNA Lysis Buffer (BioServUK) in a Class 2 Microbiological Safety Cabinet.

Patient samples were then plated in batches of 92, accommodating four wells that are required for control samples. Using a handheld barcode scanner, a barcode on a KingFisher 96 deep well plate (ThermoFisher Scientific) and anonymized barcodes (container IDs) on each neutralised sample were registered in the command centre. Neutralized samples were placed into custom 3D printed SBS format racks for the Biomek i5 liquid handling robot (Beckman Coulter). A volume of 500 μL was transferred from sample tubes to the registered 96 well plate using a Biomek i5 Span 8 liquid handler (Beckman Coulter).

Pooling and analysis were initiated by registering the plate in which pooled samples would be contained. The registered barcodes on multiple sample plates were scanned along with a new KingFisher 96 deep well plate (ThermoFisher Scientific). Subsequently, 50 μL of sample from each sample plate was transferred to the pooling plate using a CyBio FeliX liquid handler (Analytik Jena). A total pooled sample of 500 μl was used for RNA extraction using the Maxwell HT Viral TNA kit (Promega) on the CyBio FeliX liquid handler (Analytik Jena) with an elution volume of 50 μl using a custom extraction protocol ^16^. Subsequent RT-qPCR was performed using the TaqPath ^TM^ COVID-19 CE-IVD RT-PCR Kit (ThermoFisher Scientific) according to the manufacturer’s instructions and thermal cycled on a qTower3 (Analytik Jena).

Results from RT-qPCR were processed using the pooling command centre analysis capabilities, resulting in two exportable outputs of deconvoluted data on a per-sample basis: All negative patient results (in a LIMS-compatible format) and a picklist of positives with instructions (in a Biomek i5-compatible format) for rearraying samples from their original 96 well plates. A volume of 360 μL was transferred from all positive wells to a KingFisher 96 deep well plate (ThermoFisher Scientific, pre-registered in the command centre) using a Biomek i5 Span 8 liquid handler (Beckman Coulter). The retest plate was used for RNA extraction using the Maxwell HT Viral TNA kit (Promega) on the CyBio FeliX liquid handler (Analytik Jena) with an elution volume of 50 μl using a custom protocol ^16^. Subsequent RT-qPCR was performed using the TaqPath ^TM^ COVID-19 CE-IVD RT-PCR Kit (ThermoFisher Scientific) according to the manufacturer’s instructions and thermal cycled on a qTower3 (Analytik Jena). Final results were processed in the command centre and exported in a LIMS compatible format for reporting.

## Results

### Impact of pooling on analytical sensitivity

**Figure 1.**
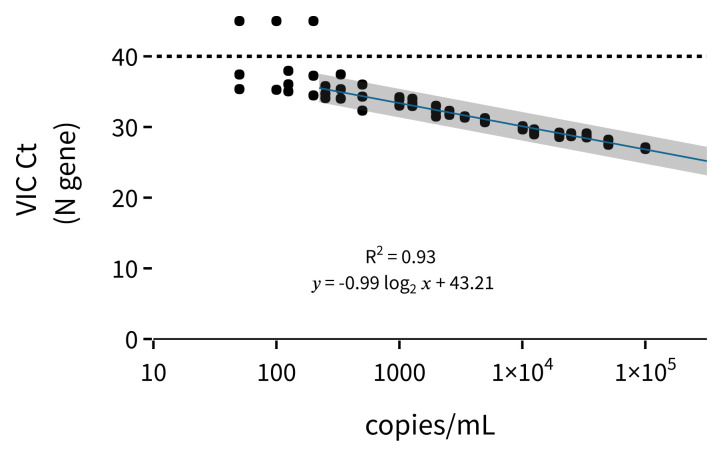
Standard curve of end-to-end RNA extraction and RT-qPCR with concentrations from 100 000 copies/mL to 50 copies/mL of MS2 sVLP containing the N gene. Each concentration has 3 technical replicates. Dotted line shows the cycle limit of detection. Line of best fit is fit to the Ct values from 250 copies/mL to 100 000 copies/mL. Error is the 95% confidence interval.

In order to address the impact of pooling on analytical sensitivity, we used our previously characterised MS2 synthetic viral-like particles (sVLPs) containing the SARS-CoV-2 N gene ^16^. By performing end-to-end RNA extraction-PCR analyses of different sVLP concentrations, we were able to analyse the lower limit of detection (LLOD) ( Figure 1). Using the N gene primer set (VIC channel) of the ThermoFisher Scientific COVID-19 assay, we showed that for our sVLP reference material, the LLOD was approximately 250 viral copies/mL. We also observed a linear response between 100 000 and 250 copies/mL ( Figure 1) and the deviation from the expected Ct ( Table 1) was always less than 1 Ct cycle.

**Table 1.** The mean Ct is the mean of three technical replicates. Expected Ct assumes that halving starting concentration should increase the Ct value by 1.

copies/mL	mean Ct	expected Ct	expected Ct - mean Ct
100017	26.99	26.99	0
50008	27.95	27.99	0.04
33292	28.77	28.58	-0.19
24933	28.86	28.99	0.13
19975	28.92	29.31	0.39
12467	29.32	29.99	0.67
10058	29.83	30.3	0.47
4958	30.98	31.32	0.34
3400	31.42	31.87	0.45
2550	31.92	32.28	0.36
1983	32.21	32.65	0.44
1275	33.46	33.28	-0.18
1000	33.73	33.63	-0.1
500	34.21	34.63	0.42
333	35.6	35.22	-0.38
250	34.86	35.63	0.77

Using the MS2 sVLP standard curve and LLOD, we were able to predict the effects of various pooling strategies on sensitivity ( Table 2). For three different sVLP concentrations, and 6 different simulated pool sizes from 1-IN-2 (equivalent to having two samples in the same pool) to 1-IN-10, we demonstrate how the analytical sensitivity translates into a practical pooling strategy. For example, with an individual sample with 1000 copies/ml, we were not able to detect the N-gene in 1-IN-5, 1-IN-8 and 1-IN-10 simulated pools ( Table 2). These values were selected because they are the most practical pool sizes that could be implemented in a diagnostic laboratory context, given turnaround time constraints and availability of liquid handlers ^17^.

**Table 2.** Mean Ct values from the VLP standard curve used to determine the sensitivity impact depending on the proposed pooling strategy. Copies/mL is the concentration of the positive sample in the simulated pool. Neat shows the Ct value without dilution and the following columns show the effect of a serial dilution (simulating a positive sample in a pool of negatives). Below 250 copies/mL there is no longer reliable detection of the target (not detected in all technical replicates).

copies/mL	Neat	1-IN-2	1-IN-3	1-IN-4	1-IN-5	1-IN-8	1-IN-10
100017	26.99	27.95	28.77	28.86	28.92	29.32	29.83
	Detected	Detected	Detected	Detected	Detected	Detected	Detected
10058	29.83	30.98	31.42	31.92	32.21	33.46	33.73
	Detected	Detected	Detected	Detected	Detected	Detected	Detected
1000	33.73	34.21	35.6	34.86			
	Detected	Detected	Detected	Detected	Not Reliably Detected		

### Impact of prevalence on pooling size

The prevalence of SARS CoV-2 in the population to be tested using pooling, has a significant impact on the pooling strategy. With increasing prevalence, the number of positive pools increases, reducing the efficiency of the pooling process. Local prevalence will vary and the decision on pool size will need to be a dynamic one, informed not only by SARS CoV-2 prevalence but also assay sensitivity and specific local laboratory constraints. A suggested approach is to determine prevalence based on a local rolling average of the preceding 7–10 days ^14^ with PHE suggesting consideration of pooling when the prevalence is less than 10% ^15^.

Analysis (incorporating the model described by Regen *et al.* ^18^) was undertaken to estimate the optimal pool size based on prevalence in a conventional modular extraction/qPCR plate system. Turnaround requirements are such that tested pools should report mostly negative results, with the number of positive samples requiring retest not exceeding the size of one subsequent extraction and qPCR run (in a plate-based format this amounts to a maximum of 92 samples). On this basis, the prevalence was used to estimate the average number of potentially positive pools, allowing calculation of the expected number of samples that would require retest (see Table 3). Whilst pools of 20 and 15 samples are unlikely due to loss in analytical sensitivity, they were included for completeness.

**Table 3.** Estimated optimal pool size based on prevalence.

Prevalence range	Number of samples per pool	Number of samples per pooled plate	Average number of positive samples that will need retest per pool	Average number of positive samples that will need retest per plate
**0.1-0.2%**	20	1880	2-4	40-80
**0.3-0.4%**	15	1410	5-6	75-90
**0.5-0.9%**	10	940	5-9	50-90
**1.0-1.4%**	8	752	7-11	56-88
**1.5-2.6%**	6	564	9-15	54-90
**2.7-3.8%**	5	470	13-18	65-90
**3.9-6.1%**	4	376	15-23	60-92
**6.2-10.9%**	3	282	18-31	54-93
**11-24.4%**	2	188	21-46	42-92

### Impact of pooling on capacity

Pooling offers the possibility of increasing overall testing capacity by increasing analyser capacity with the introduction of some extra pooling specific steps to the existing laboratory process. For significant increases in testing capacity to be realised, the entire workflow needs to be optimised. While pooling addresses an analyser capacity bottleneck it will not provide capacity benefits if the limiting factor sits elsewhere in the process (e.g. limited safety cabinets for sample neutralisation). Each laboratory will have its own particular set of challenges to overcome and potential capacity improvements will be variable based on the local context.

Two different pooling workflow scenarios were considered, namely an independent pooling workflow and an integrated pooling workflow. Both provide distinct advantages depending on the local analyser infrastructure.

**Figure 2.**
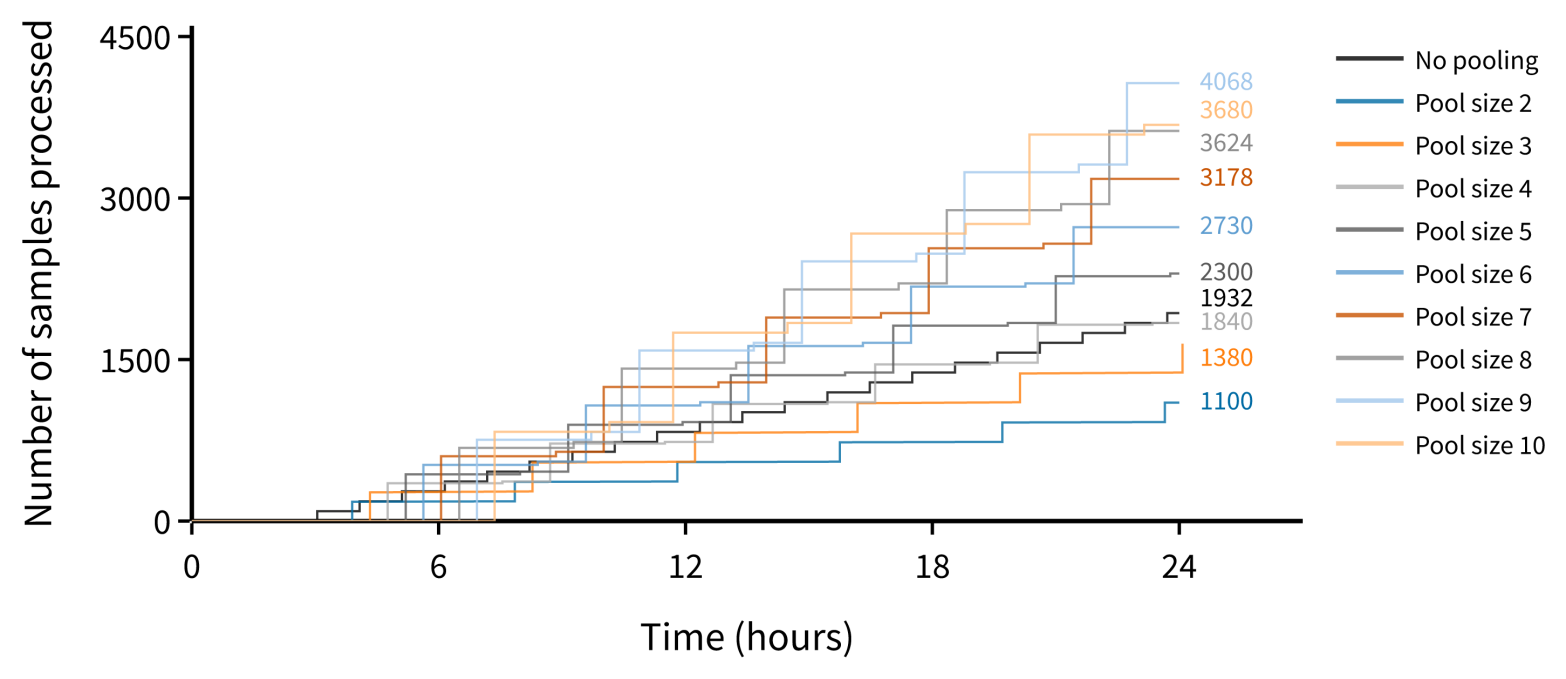
Estimated number of samples processed using a single analyser in a 24-hour period (where analyser capacity is the limiting factor).

(1) *Independent pooling workflow* - In this workflow all requirements for pooling, namely testing of pooled and retest samples, are done as part of an independent workflow (i.e. not feeding into the routine testing workflow). As such both steps are completed before the cycle can be repeated. In order to estimate the potential capacity impact achievable by implementation of an independent pooling workflow, the local testing process was modelled based on the following assumptions : (i) prevalence levels of 0.9%; (ii) adequate resources were available to address other bottlenecks in the sample processing; (iii) a single analyser platform was available (Analytik Jena FeliX for extraction and Analytik Jena qTower ^3^ for amplification) and that both the pooled plate and retest plate were run on the same analyser. A time analysis of the steps in the process is shown in the Methods (Methods Table 1). Although this simple model is limited, as it only considers a single platform for analytical processing, it is useful to illustrate the potential capacity impact of pooling in the following scenarios:

Scenario A - Analyser capacity is the limiting factor. In this scenario, any pre-analytical bottlenecks have been resolved with a single extraction / amplification workflow testing both the pool and retest plates (with no reagent supply constraints). For a pool size of 10, we estimate that the projected upper limit of capacity for routine testing increases twofold ( Figure 2). 

**Figure 3.**
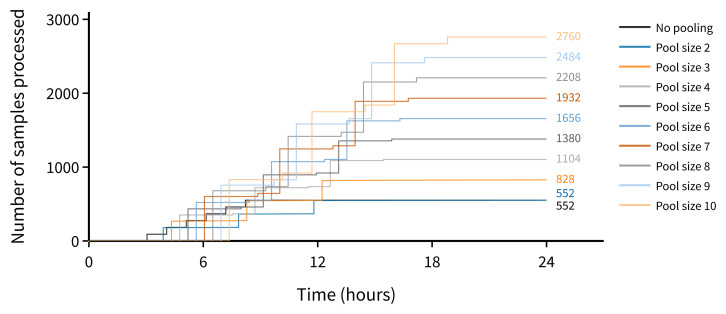
Estimated number of samples processed using a single analyser in a 24-hour period (where analyser is limited to 6 runs a day by reagent shortage).

Scenario B - Analyser capacity is constrained. In this scenario the assumptions are similar to scenario A except that the number of runs on a single analyser are limited (for example as a result of reagent supply issues). Using a pool size of 10, we estimate that the projected upper limit of capacity increase for routine testing is fivefold ( Figure 3).

*(B) Integrated pooling workflow.* In this workflow, only the pooled samples are tested through the pooling workflow with retesting of individual samples feeding into the standard testing workflow. This allows more rapid cycling through the testing of pooled plates but requires sufficient capacity in the standard workflow to test samples from the positive pools. We would anticipate that the majority of laboratories would adopt this approach. In order therefore to estimate the capacity benefit of this model we used an equation where the increase in capacity is equal to the total number of samples divided by the sum of the number of tests used to tests pools plus the number of tests for retesting (Methods, Equation 1).

The prevalence and pool size will be key determinants on any capacity increase. For example, in the setting where there are no positive pools, the magnitude of the potential capacity increase will be the pool size multiplied by the number of tests available for pooling (i.e. the theoretical maximum capacity benefit). As prevalence increases the capacity benefit will decrease. Assuming a scenario where 100 tests are available for pooling, the capacity increase by pool size and prevalence is provided in Table 4.

**Table 4.** Estimated capacity increase by pool size and prevalence.

Prevalence	Pool Size	Tests available	Number of samples tested	Maximum Number of positive pools	Number of initial negative samples reported	Number of samples retested	Capacity increase (number of extra samples tested)	Capacity increase (as multiple of baseline)
10%	2	100	200	20	160	40	60	**1.43**
	3	100	300	30	210	90	110	**1.58**
	4	100	400	40	240	160	140	**1.54**
	5	100	500	50	250	250	150	**1.43**
	6	100	600	60	240	360	140	**1.30**
	7	100	700	70	210	490	110	**1.19**
	8	100	800	80	160	640	60	**1.08**
	9	100	900	90	90	810	-10	**0.99**
	10	100	1000	100	0	1000	-100	**0.91**
5%	2	100	200	10	180	20	80	**1.67**
	3	100	300	15	255	45	155	**2.07**
	4	100	400	20	320	80	220	**2.22**
	5	100	500	25	375	125	275	**2.22**
	6	100	600	30	420	180	320	**2.14**
	7	100	700	35	455	245	355	**2.03**
	8	100	800	40	480	320	380	**1.90**
	9	100	900	45	495	405	395	**1.78**
	10	100	1000	50	500	500	400	**1.67**
1%	2	100	200	2	196	4	92	**1.92**
	3	100	300	3	291	9	182	**2.75**
	4	100	400	4	384	16	268	**3.45**
	5	100	500	5	475	25	350	**4.00**
	6	100	600	6	564	36	428	**4.41**
	7	100	700	7	651	49	502	**4.70**
	8	100	800	8	736	64	572	**4.88**
	9	100	900	9	819	81	638	**4.97**
	10	100	1000	10	900	100	700	**5.00**

### Reagent and labware utilisation

In order to explore the potential reduction in testing resources for pooling, we calculated usage of plasticware, tips and testing reagents using different pool sizes using our modular plate-based workflow. We found significant savings in all resource requirements with up to 45% reduction in tips, 76% reduction in plates and 90% reduction in both RNA extraction and qPCR reagents ( Table 5). These significant reductions in resource requirements are particularly pertinent for diagnostics laboratories in national testing programs, where acute shortages of reagents and plastics during the pandemic have caused decreases in testing capacities.

**Table 5.** Potential reduction in testing resources for different pool sizes.

Pool Size	1	2	3	4	5	6	7	8	9	10
**Plastics**										
**1 mL tips Percent Reduction compared to standard test**	0%	1%	13%	18%	22%	24%	26%	27%	28%	28%
**10-200μL tips Percent Reduction compared to standard** ** test**	0%	50%	67%	75%	80%	83%	86%	88%	89%	90%
**Total Tips**	**0%**	**15%**	**27%**	**34%**	**38%**	**40%**	**42%**	**43%**	**44%**	**45%**
**Deep well plates Percent Reduction compared to** ** standard test**	0%	29%	48%	57%	63%	67%	69%	71%	73%	74%
**PCR Plates Percent Reduction compared to standard test**	0%	50%	67%	75%	80%	83%	86%	88%	89%	90%
**Total Plates**	**0%**	**31%**	**50%**	**59%**	**65%**	**69%**	**71%**	**73%**	**75%**	**76%**
**Reagents**										
**Extraction Percent Reduction compared to standard test**	0%	50%	67%	75%	80%	83%	86%	88%	89%	90%
**qPCR reagents Percent Reducation compared to** ** standard test**	0%	50%	67%	75%	80%	83%	86%	88%	89%	90%

### Staffing and infrastructure requirements

While specific requirements for the implementation of pooling will differ between laboratories, generally three different high throughput methods for SARS-CoV-2 testing are employed:

(1) Separate extraction followed by qPCR setup and qPCR measurement performed on 96 well plates (plate-based)

(2) End to end sample in result out system using batches of samples for parallel testing (e.g. Roche 6800, Abbott M2000)

(3) End to end sample in result out system with samples being tested randomly (e.g. Panther, Alinity M)

Here we will only address workflows for 1 and 2 (which were available in the local context) and assess how pooling fits within these workflows, and how the existing infrastructure can be augmented to maximise available resources, and address capacity and consumable constraints. In Table 6, we describe the additional staff and infrastructure required to double a labs capacity of 2500 samples a day for both workflows. This detail is based on the precise mapping of the entire process (available on Zenodo). Most of the additional staff are required for the pre-analytical sample processing steps which still remains the major bottleneck for scaling testing. Further staff time is required for pooling the primary samples into suitable formats for each testing workflows. After this step, we envisage no further staff time requirements above the normal levels required for testing. Additional infrastructure is required by both workflows for pre-analytic sample processing (bench space and class-I cabinets) and primary pooling of samples (automated liquid handling robots). For plate pooling an additional 96 tip head robot is required ( Table 6). We have also identified a key need for sample tracking software that would link the primary tube barcode to the pooled result and allow retesting of those samples in a positive pool.

**Table 6.** Additional staffing and infrastructure requirements to double capacity of a lab currently processing 2500 samples a day.

Area	Required additional Staff/Infrastructure		
	Infrastructure for plate-based workflows	Infrastructure for batch based end-to- end workflows	Additional Staff (2 shifts per day 24/7)
**Pre-analytic: samples arrive**			..
**Pre-analytic: sample booking**	**Additional sample booking stations and bench space** for booking samples into LIMS		6
**Pre-analytic: primary sample** ** processing**	**Additional class-I cabinet** for sample neutralisation and swab removal and racking **Additional bench space** to store racked samples		4
**Pooling: primary tube transfer**	**Additional automated liquid handling robot** with barcode reader and at least 8 tip head for transfer from primary tubes to pool tubes OR **Use of other readily available automation** such as ELISA DS2 liquid handling robot to transfer from primary tubes to low profile plates and capture tube barcode.		4
**Pooling: plate pooling**	**Additional robotic 96 tip head** for plate- to-plate transfer (10 minutes per pool, up to 10 samples per pool) OR **Manual Pipetting** (40 minutes per pool)	NA	..
**Pooling: sample tracking software**	**Software** to link the primary tube barcode to pool result.		..
**Testing: extraction**	Existing testing allocation	Existing Roche 6800 testing allocation	..
**Testing: amplification**			..

### Development of a deployable software platform to enable pooling

Deconvoluting and re-arraying positive pools required a novel software solution, as traditional LIMS-based and spreadsheet-based approaches are insufficient in terms of core capabilities and data integrity, respectively. To manage these requirements, a custom cloud-based web application was developed using the Riffyn Nexus ^TM^ software platform as its core, which enabled usage of Riffyn’s patented data integration algorithm.

**Figure 4.**
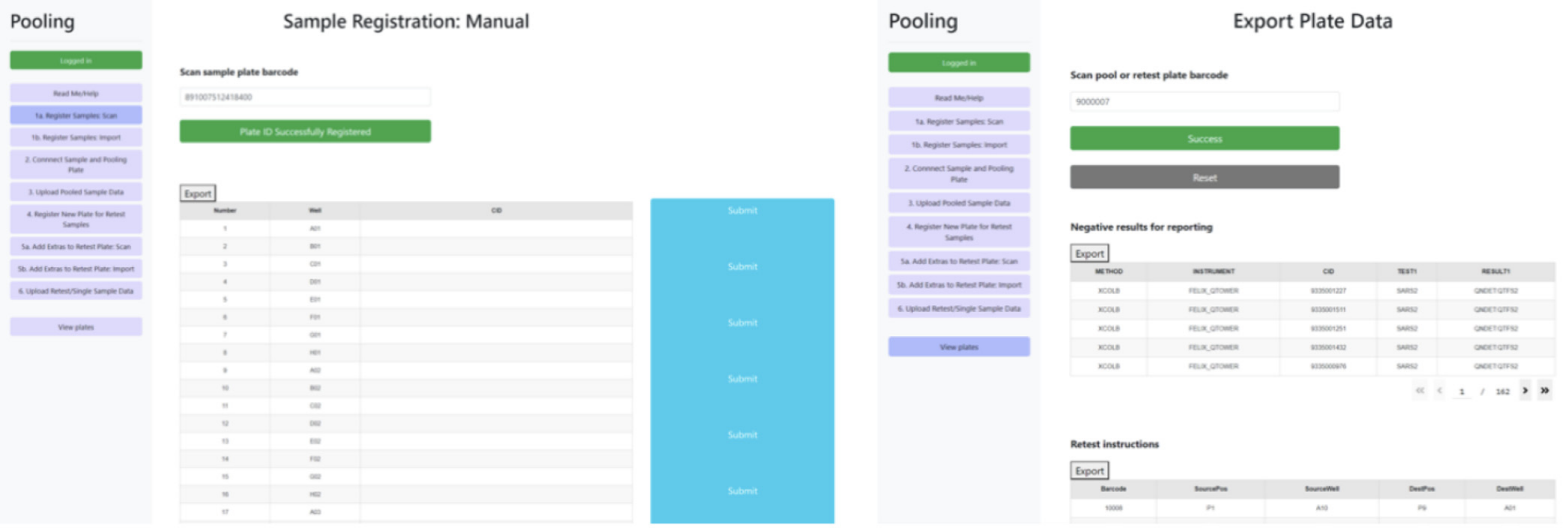
Screen shots of the pooling software, illustrating container ID (sample barcode) scanning and registration of sample plates (left) and export of qPCR processing (right).

The comprehensive solution was engineered to integrate with laboratory operations to capture and share data ( Figure 4). Anonymised container IDs (generated within the laboratory LIMS system) and plate barcodes were registered via simple barcode scans of plated individual and pooled samples. Results from qPCR analyses were processed after a simple drag-and-drop CSV file upload, generating two CSV exports to support the pooling workflow: (1) instructions for the liquid handler for re-arraying pooled samples that returned a positive or inconclusive result; and (2) a LIMS-compatible CSV-format file with all deconvoluted, individual negative results.

### Technical feasibility of pooled testing

**Figure 5.**
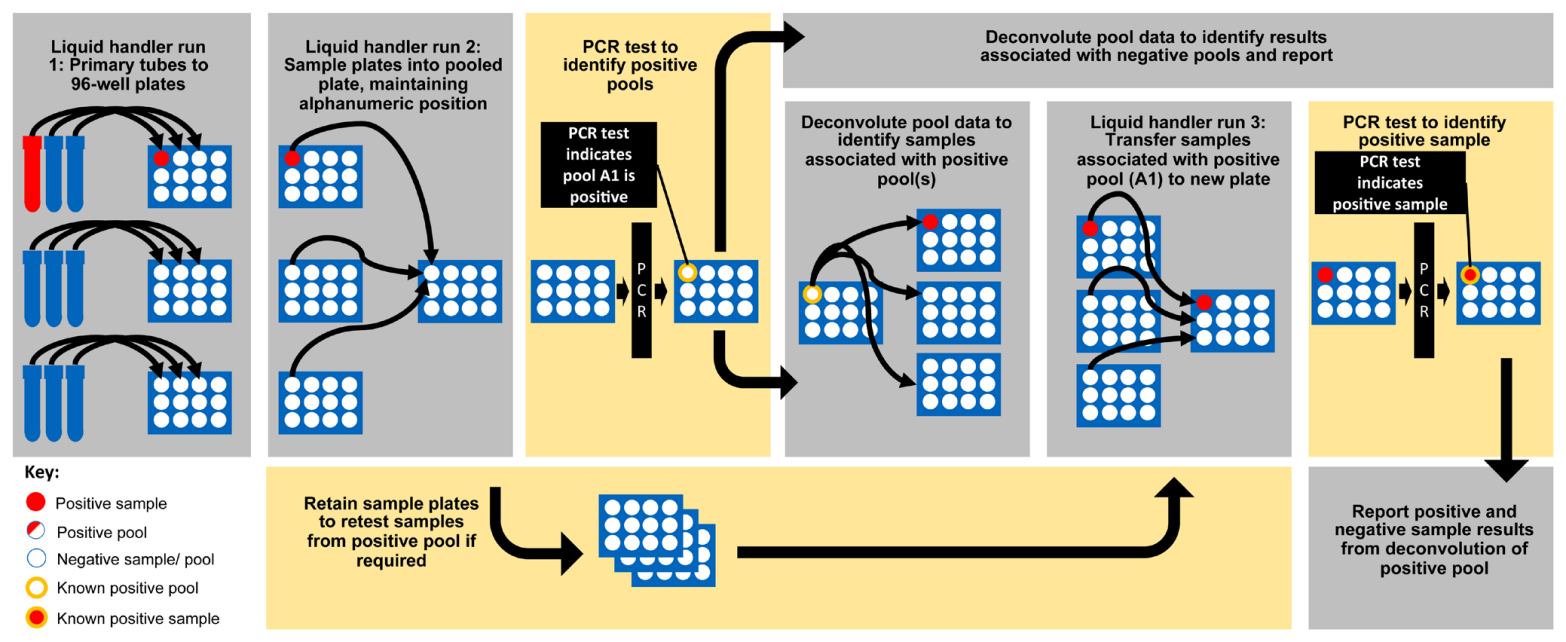
The designed and implemented pooling workflow for plate-based SARS-CoV-2 testing.

In order to assess the technical feasibility of the designed pooling workflow, nine hundred and twenty (920) patient samples were booked into North West London Pathology’s Sunquest LIMS test environment. The samples were then run through the pooling workflow ( Figure 5) and final results were reported to the Sunquest LIMS test environment.

**Figure 6.**
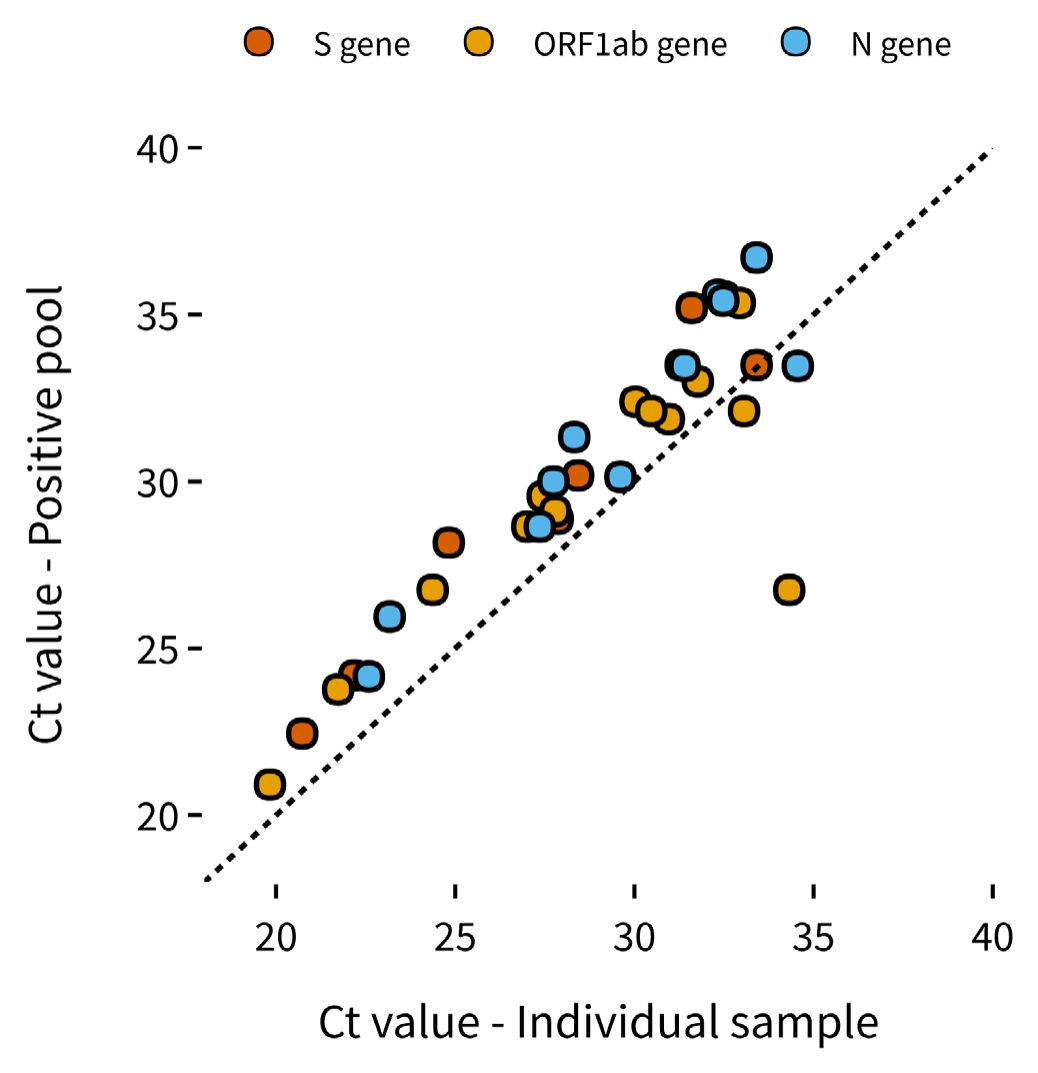
Positive samples: Ct values when tested individually versus Ct of respective positive pool for all 3 assay targets (S gene, ORF 1ab and N gene). Sample Ct values found to the right and below the dotted line were found in pooled wells with other positive samples with higher viral loads.

The results are shown in Figure 6 and Table 7, and on average pooling resulted in a loss of analytical sensitivity of 2.335 cycles in the ABY channel (S gene), 1.74 cycles in the FAM channel (ORF1ab) and 2.3 cycles in the VIC channel (N gene). Using our sensitivity analysis with our sVLPs, Sample 3 was at around the reliable N gene limit of detection (~467 copies) in our retest, and therefore would usually not be detectable in a 1-IN-10 pool. However, Sample 3 was still detected using our pooling strategy which could be due to the other gene targets of the ThermoFisher assay having lower a LLOD than that calculated by our N-gene sVLPs. In summary, we observed an expected increase in Ct value with pooled testing, however, due to our multi-target assay we were still able to detect samples with concentrations of below 1000 copies/mL.

**Table 7.** Summary of positive results from 92 pools of 10 samples and the subsequent deconvolution and retesting of positive pools.

Positive Samples	Pooled Sample Well	Pooled Sample Ct Values				Retest Sample Well	Retest Sample Ct Values			
		ABY	FAM	VIC	JUN		ABY	FAM	VIC	JUN
**Sample 1**	A10	35.54	33	35.59	27.01	D09	32.51	31.77	32.33	26.32
**Sample 2**	A11		29.56	31.33	25.75	D10		27.44	28.32	25.28
**Sample 3**	B02		35.35		26.79	F10	34.13	32.94	34.64	25.85
**Sample 4**	B03	28.17	26.75	28.65	25.98	D12	24.82	24.37	27.36	26.12
**Sample 5**	B03	28.17	26.75	28.65	25.98	F11		34.32		26.97
**Sample 6**	B06	30.18	29.11	30.14	26.22	G01	28.43	27.79	29.61	26.88
**Sample 7**	B09	35.2	31.87	36.71	26.66	E02	31.6	30.96	33.41	26.14
**Sample 8**	C05		32.39	35.42	26.41	E03		30.03	32.48	26.15
**Sample 9**	D12	33.49	32.11	33.46	26.22	E04	31.29	30.47	31.4	26.47
**Sample 10**	D12	33.49	32.11	33.46	26.22	D05	33.41	33.06	34.56	26.67
**Sample 11**	E07	28.88	28.65	30	27.1	E05	27.86	27.01	27.74	26.22
**Sample 12**	G06	22.45	20.92	24.16	25.2	E06	20.73	19.83	22.59	27.52
**Sample 13**	H01	24.19	23.77	25.95	28.26	E07	22.18	21.73	23.17	28.33

## Discussion

Here we describe a proof-of-concept study on the implementation of a plate-based pooling strategy for SARS-CoV-2 testing in an NHS diagnostic laboratory. We systematically examined the scientific, technical and operational constraints of implementing pooling in any frontline diagnostic laboratory. We not only considered the technical challenges in sample tracking of positive samples within pools and accompanying liquid handing, but modelled resources savings and analyser capacity increases and considered operational constraints such as additional infrastructure and staff requirements. 

Group testing is an obvious proposition to increase testing capacity in reagent and analyser constrained settings. However, simpler solutions, such as swab pooling ^19^, are not viable options for diagnostic laboratories where turnaround times preclude follow up retesting. Therefore, laboratory-based pooling, with its accompanying logistical and operational challenges remains the only viable option. Given that each laboratory has its own specific requirements, we focused on creating a generalisable approach that could be implemented elsewhere. We developed generalisable laboratory automation workflows and easily deployable software and our operational models can be altered based on assumptions relevant to a local laboratory context.

While we focused on plate-based pooling in this work, we also considered the use of tube-based end-to-end solutions (such as the Roche 6800). These platforms have already been approved for pooling of up to 6 samples per pool ^12^ and many principles are shared between plate-based and tube-based pooling. We found that given the limited availability of Roche reagents worldwide, an optimal pooling approach could make use of the Roche allocation for pooling, with retest samples tested on a different platform to preserve test capacity on the instrument with limited supplies. Given a fixed allocation of tests for an analyser, this would substantially increase the number of samples that could be tested ( Figure 3).

There are limitations to our study, pooling of samples leads to the loss of a sample adequacy control which can lead to false negative results. Our laboratory uses an assay that uses a spike-in processing control which would control for severe inhibition of the reaction. Nevertheless, this is one disadvantage of pooling that should be considered before implementation. Pooling would usually only be implemented when there are resource constraints that make it the most viable option to keep a diagnostic service running or when there are clear advantages over testing samples individually.

With the roll out of the vaccine, decreasing disease prevalence and a growing interest in surveillance for variants of concern, we see laboratory-based pooling as being an attractive option. Pooling will not only optimize the use of testing resources including the use of reagents and labware but also increase capacity for the testing of asymptomatic staff and patients. Here we have shown that laboratory-based pooling is scientifically, technically and operationally feasible in an NHS diagnostic laboratory. We believe that this work provides a generalisable approach for other NHS diagnostic laboratories to implement adaptive pooling strategies in their own specific settings.

## Data availability

### Underlying data

Zenodo: Rapid design and implementation of an adaptive pooling workflow for SARS-CoV-2 testing in an NHS diagnostic laboratory: A proof-of-concept study, https://doi.org/10.5281/zenodo.5542412 ^17^.

This project contains the following underlying data:

- design_standardcurve.xlsx
- Detailed process map for plate-based pooling developed by NWLP and Imperial College.pdf
- rawdata_pooling_pooledplate.csv
- rawdata_pooling_retest_plate1.csv
- rawdata_pooling_retest_plate2.csv
- rawdata_standardcurve.csv

Data are available under the terms of the Creative Commons Attribution 4.0 International license (CC-BY 4.0).

More detailed data, including that uploaded to the LIMS test environment, cannot be provided as although it contains anonymised container IDs, these can be linked back to patients if someone has access to the laboratory LIMS environment.

## Ethics statement

Surplus clinical material was used to validate the assay as per normal practice and does not require ethical review.
