# Supplementary material for: Rapid design and implementation of an adaptive pooling workflow for SARS-CoV-2 testing in an NHS diagnostic laboratory: a proof-of-concept study
Source: Wellcome Open Res. 2021 Oct 13;6:268. [Version 1] doi: 10.12688/wellcomeopenres.17226.1 (PMC8591522; doi:10.12688/wellcomeopenres.17226.1)
Supplement: Peer review history

# Peer review: Reviewer response for version 1

**DOI:** 10.21956/wellcomeopenres.19037.r46676

**Published:** 2021-11-11

**Authors:** Nei-Yuan Hsiao

**Affiliations:** 1 Institute of Infectious Disease and Molecular Medicine, University of Cape Town, Cape Town, South Africa; 2 National Health Laboratory Service, Johannesburg, South Africa

In this proof of concept study designed in the UK NHS environment, Crone and colleagues described the impact of pooled NAT approaches on sensitivity, efficiency and capacity of high-throughput SARS-CoV-2 molecular testing while demonstrating its feasibility and merit. The authors assessed the many important factors associated with the pooling approach of SARS-CoV-2 testing including scientific, technical and operational. The methods that outlined the feasibility, both conceptual and physical, are appropriately designed and well implemented, with detailed and rigorous analyses. I found the description of work conducted can be inaccessible at times due to unfamiliarity with jargon and knowledge with specific automation platforms but overall the authors were able to accurately communicate the complexity around pooled NAT in the setting of SARS-CoV-2 testing with variable population prevalence and laboratory constraints.

 The work is entirely based on the assumption that automation is available and unfortunately does not tackle the feasibility of pooling in a low-resource setting where pooling needs to be done by hand. In the analyses, the impact of contamination and assay failure rate was also not considered. These factors may be highly context-dependent and thus may be beyond the scope of research. It would be useful to include these considerations in the study limitation section of the discussion, as often the setting that would most likely consider pooled NAT are often those with limited resources.

 Overall the data and findings of the study are highly informative and definitely worthy of publication.

Is the work clearly and accurately presented and does it cite the current literature?

Yes

If applicable, is the statistical analysis and its interpretation appropriate?

Yes

Are all the source data underlying the results available to ensure full reproducibility?

Yes

Is the study design appropriate and is the work technically sound?

Yes

Are the conclusions drawn adequately supported by the results?

Yes

Are sufficient details of methods and analysis provided to allow replication by others?

Yes

Reviewer Expertise:

Clinical virology, diagnostic research, Molecular diagnostic, viral epidemiology

I confirm that I have read this submission and believe that I have an appropriate level of expertise to confirm that it is of an acceptable scientific standard.

# Peer review: Reviewer response for version 1

**DOI:** 10.21956/wellcomeopenres.19037.r46415

**Published:** 2021-10-25

**Authors:** Saranya Narayan

**Affiliations:** 1 Neuberg Ehrlich Laboratory, Chennai, Tamil Nadu, India; 2 JEENOMICS (NGS HLA Laboratory), Chennai, Tamil Nadu, India

The article is very comprehensive. Pooling of samples for SARS-CoV-2 testing, depending on the existing prevalence of infection and to study the impact of pooling on the analytical sensitivity of the RT-PCR assay, is the critical part of the study.

 Considerations like the impact on the workflow - whether as part of an independent (with and without reagent crunch) or an integrated pooling workflow - are often overlooked and were very clearly validated as shown by the data provided.

 Balancing staffing and infrastructure requirements on the one hand, with planning an optimum workflow with an efficient validated pooling system so as to optimise benefits on the other, together with a software that helped decode the pooling strategy (clear and concise data and figures provided) have all contributed to this beautifully planned and executed study.

 As the authors pointed out in the discussion, false-negative reports are a risk when the pooling of samples is done. Hence, implementation of this strategy can be done ONLY when the prevalence of the infection is below an acceptably low level for that population specifically. Especially in resource-poor nations, with larger populations having acquired the infection and more vaccine roll-out, this does offer a very useful method to get greater numbers of the population screened more rapidly.

Is the work clearly and accurately presented and does it cite the current literature?

Yes

If applicable, is the statistical analysis and its interpretation appropriate?

Yes

Are all the source data underlying the results available to ensure full reproducibility?

Yes

Is the study design appropriate and is the work technically sound?

Yes

Are the conclusions drawn adequately supported by the results?

Yes

Are sufficient details of methods and analysis provided to allow replication by others?

Yes

Reviewer Expertise:

Infectious Disease, immunogenetics

I confirm that I have read this submission and believe that I have an appropriate level of expertise to confirm that it is of an acceptable scientific standard.
